# Graft Failure After Revascularization for Chronic Limb-Threatening Ischaemia (CLTI) Patients: The Role of Graft Surveillance

**DOI:** 10.7759/cureus.53036

**Published:** 2024-01-27

**Authors:** Mohammad Mostafizur Rahman Miah, Dani Avabde, Isabella Ghahramani, Raehan Hemanth, Ridda Abbas, Quratulain Maha, Andrew Beech, Murtaza Salem

**Affiliations:** 1 Vascular Surgery, Nottingham University Hospitals NHS Trust, Nottingham, GBR; 2 Surgery, Nottingham University Hospitals NHS Trust, Nottingham, GBR

**Keywords:** graft occlusion, duplex scan, surveillance, risk factors, infrainguinal bypass

## Abstract

Introduction

Failure of infrainguinal bypass grafts remains a major problem tackled by vascular surgeons despite a meticulous surgical technique. All infrainguinal bypasses should go under routine surveillance to pick the grafts at risk for the prevention of graft failure.

Objectives

The aim was to find out if we were adhering to the European Society of Vascular Surgery (ESVS) guidelines in the management of chronic limb-threatening ischaemia (CLTI) patients, including postoperative follow-up and to monitor whether the patients were having postoperative duplex surveillance scans to pick any graft at risk.

Methods

All patients who underwent infra-inguinal bypass procedures for CLTI during the last eight months (from mid-January to mid-September 2023) in our vascular unit were included. Retrospective data were collected.

Results

A total of 38 patients had lower limb bypass procedures over the last eight months (from 15 January till 14 September 2023). However, two femoral-femoral (fem-fem) crossovers, one Ilio-popliteal, and one pedal bypass were excluded. Thus, a total of 36 patients were included in the study (n=34). The vast majority (n=27, 79.4%) had femoro popliteal bypass anastomosing distally to above knee (AK) or below knee (BK) popliteal artery, and the rest (n=7, 20.5%) had distal bypass (fem-distal or pop-distal bypass). Moreover, 18% of patients had amputation, 15% of patients died, and 61% of the remaining patients were on surveillance. Of those, who were not on surveillance, 44% of them had graft occlusion.

Conclusion

Surveillance can predict graft at risk, and the graft occlusion can be prevented by appropriate intervention. Every vascular unit should have its own post-procedural follow-up strategies.

## Introduction

Failure of infrainguinal bypass grafts remains a major problem tackled by vascular surgeons despite a meticulous surgical technique. The graft failure can be divided into early, intermediate, and late depending on the time of onset. Early graft failure usually occurs within < 30 days of the procedure, intermediate graft failure occurs between 30 days to two years’ time, and late graft failure occurs in >2 years of surgery [1]. If the graft failure occurs within 48 hours of the procedure, it is usually due to technical errors, such as poor anastomosis poor inflow or outflow or a retained valve cusp of the vein graft; however, if the graft fails within two to 12 days, it is usually caused by the graft thrombo-reactivity [1]. The cause of intermediate graft failure is intimal hyperplasia, which is more common in downstream at the outflow anastomosis [2]. Sometimes patients remain asymptomatic after the graft failure or thrombosis. However, often they are more symptomatic than their initial preoperative presentation. Very little has been known about the predictive value of various risk factors and the definite role of surveillance in picking up the bypass grafts at risk early in a view to offer appropriate treatment. There are a few studies that have addressed the identification of specific vascular risk factors for infrainguinal bypass graft occlusion and the role of regular postoperative follow-up [3]. Grafts at risk can be identified using clinical symptoms and non-invasive imaging, including arterial duplex ultrasound if performed postoperatively. The duplex ultrasound may be very useful in identifying the grafts at higher risk of failure, which may necessitate graft revision or endovascular intervention [4]. In a prospective study of 204 patients, it was shown that early duplex scan surveillance detects a clinically significant stenosis that needs revision or re-intervention [5]. Interterm follow-ups can identify the grafts at risk of thrombosis. Critical limb-threatening ischaemia (CLTI) is the severe form of chronic limb ischaemia with rest pain and or tissue loss where the perfusion of the limb is critically reduced to threaten the limb salvage. The European Society of Vascular Surgery (ESVS) suggests that it is a good practice to do regular surveillance after bypass graft surgery for CLTI patients [6]. Various evidence suggests that graft stenosis imposes a three-to-six-fold risk of graft occlusion if left untreated [7]. There is a lack of strong evidence in the form of a double-blinded prospective randomised control trial; however, there are retrospective studies to demonstrate the superiority of intervention in asymptomatic patients with graft stenosis [8]. If the bypass graft fails, amputation may be necessary.

This study sheds light on several crucial elements in the management of CLTI following infrainguinal bypass surgery. The pronounced prevalence of traditional vascular risk factors such as smoking, hypertension, and diabetes mellitus in our patient cohort emphatically underscores the critical importance of rigorous risk factor modification. These factors have been well-documented to contribute substantially to the likelihood of graft failure and to the general deterioration of vascular health. Moreover, the results unveiled a concerning disparity in postoperative surveillance practices. While a subset of patients was actively enrolled in a graft surveillance program, a significant proportion was not, which could have profound implications for the timely detection of graft complications. The vital role of diligent surveillance is further underscored by the observed lower rates of graft occlusion among patients who were consistently monitored. Additionally, the study highlights the critical importance of selecting the appropriate graft material. The preference for autogenous venous grafts, when available, is consistent with current evidence, indicating superior long-term patency rates in comparison to synthetic grafts. This selection is particularly crucial in light of the high burden of comorbid conditions present in our patient population. The observed mortality and major limb amputation rates, while aligning with existing literature for this high-risk demographic, underscore the pressing need for more aggressive and multifaceted management strategies. These should encompass a comprehensive array of medical and surgical interventions, augmented by patient education and the implementation of more thorough follow-up protocols.

The primary aim of this comprehensive study was twofold, focusing on two pivotal aspects:

Adherence to ESVS guidelines: The study meticulously evaluated adherence to the ESVS guidelines in the postoperative management of patients who underwent infrainguinal bypass surgery for CLTI. This detailed assessment aimed to determine the extent to which established standard protocols, especially in the realms of postoperative follow-up and meticulous monitoring, were being rigorously adhered to in clinical practice.

Monitoring postoperative outcomes: A key aim was to scrutinize the effectiveness of the implementation of postoperative duplex surveillance scans in the detection of grafts at heightened risk of failure. This aspect of the study sought to understand the extent of the utilization of these scans in routine patient follow-up and to correlate their diagnostic accuracy with actual clinical outcomes, including graft patency, limb salvage rates, and overall patient survival rates.

Finally, this study highlights the intricate complexity involved in managing CLTI patients post-infrainguinal bypass. It underscores the paramount importance of strict adherence to established guidelines, the necessity of thorough and regular follow-up, and the development of individualized care plans tailored to improve patient outcomes. This multifaceted approach should be a cornerstone priority for vascular units tasked with managing this challenging patient demographic.

## Materials and methods

Study design

This investigation employed a retrospective cohort study design to achieve its aims, ensuring a thorough and comprehensive analysis of the data. The study was conducted within a single, specialized Vascular unit and included all patients who underwent infrainguinal bypass procedures for CLTI over a specified eight-month period (from mid-January to mid-September 2023), providing a contemporary insight into current clinical practices.

Inclusion and exclusion criteria

Patients who had an infrainguinal bypass procedure for CLTI within the study timeframe and patients who had sufficient follow-up data available were included. Exclusion criteria were established for those patients who underwent femoral-femoral (fem-fem) crossover, Ilio-popliteal, and pedal bypass surgeries and those patients with incomplete medical records or insufficient follow-up data, ensuring a focused and relevant patient cohort.

The study meticulously selected a specific patient demographic to ensure both the relevance and specificity of its findings. This study focused exclusively on patients who underwent an infrainguinal bypass procedure for CLTI within the specified study period (mid-January to mid-September 2023), providing a clear and focused patient group for analysis. The study specifically included those who received infrainguinal bypass surgeries, maintaining a concentrated focus on this particular therapeutic approach and its outcomes. Including patients who had their surgeries performed within the specified eight-month period allowed for a recent and relevant overview of clinical practices and outcomes, providing contemporaneous insights into the treatment of CLTI. Only patients who had sufficient follow-up data available, particularly those who had undergone postoperative evaluations and duplex scanning as per the study protocol, were included, ensuring the reliability and comprehensiveness of the study's findings.

To ensure the focus and integrity of the study, certain groups were systematically excluded. Patients who underwent fem-fem crossover, Ilio-popliteal, and pedal bypass surgeries were excluded due to the differing clinical courses and outcomes associated with these procedures compared to infrainguinal bypass surgeries. Patients with incomplete medical records or insufficient follow-up data were excluded to maintain the accuracy and reliability of the study's findings.

Data collection

The study collected comprehensive data on demographic factors, medical history, vascular risk factors, surgical details, postoperative medications, and follow-up. Detailed analysis of age and gender distributions, in-depth examination of comorbidities such as ischemic heart disease (IHD) and chronic renal failure (CRF), and assessment of smoking status, hypertension, and diabetes mellitus were performed. Evaluation of the site of distal anastomosis (popliteal artery above or below knee vs. crural or pedal artery) and the type of graft material used (autologous vein, prosthetic, homogenous, or composite graft) were also done. Comprehensive documentation of post-surgical medication regimes was noted. Rigorous evaluation of patients at predetermined intervals post-operatively, focusing on clinical assessment (including interval history and pulse examination) and duplex scanning to assess graft patency.

The study focused on measuring key outcomes such as graft patency, the incidence of graft failure, the necessity for subsequent interventions, limb salvage rates, and mortality.

Data analysis

The data were meticulously analyzed to discern patterns and correlations between adherence to ESVS guidelines and postoperative outcomes. The study also evaluated the predictive effectiveness of duplex surveillance scans in identifying potential graft failures.

Employing this robust methodological approach, the study aimed to provide a thorough overview of the current practices in managing CLTI post-infrainguinal bypass and to identify key areas where patient care and outcomes can be significantly improved.

## Results

The results of the study provided a rich and insightful dataset regarding the outcomes of infrainguinal bypass procedures in the context of CLTI management.

Patient demographic

In the beginning, a total of 38 patients were considered. However, 34 patients were included post-exclusion criteria application, and 29.4% (n=10) of the cohort were female. The patient age spectrum ranged from 55 to 87 years, averaging around 70.75 years. The prevalence of ischemic heart disease stood at 23.5% (n=8), with chronic renal failure present in 8.8% (n=3) of the patients (Table 1).

**Table 1:** Showing the demographics, risk factors, procedural details, and postoperative antiplatelets and/or anticoagulants used. Fem-pop (femoro popliteal), Fem-distal (femoro distal), AK (above knee), BK (below knee), AT (anterior tibial), PT (posterior tibial), TPT (tibio-peroneal trunk), GSV (great saphenous vein)

Variables	Total Number of Patients (n=34)	Fem-Pop (n=27, 79.41%)	Distal (n=7, 20.59%)
	Range or % or Mean	% or Mean	% or Mean
Demographic factors			
Female gender	n=10, 29.4%	n=8, 29.6%	n=2, 28.6%
Age	55-87y, Mean 70.75y	Mean 71.85y	Mean 71.57y
Medical History			
Ischemic heart disease	n=8, 23.5%	n= 6, 22.22%	n=2, 28.6%
Chronic renal failure	n=3, 8.8%	n=3, 11.11%	n=0
Vascular Risk factors			
Current Smoker	n=18, 52.9%	n=14, 51.85%	n=3, 42.86%
Hypertension	n=24, 70.5%	n=21, 77.78%	n=3, 42.86%
Diabetes mellitus	n=15, 44.1%	n=11, 40.74%	n=4, 57.14%
Distal anastomotic site for Fem-pop and Fem-distal bypass		BK n=23, 85.19%	AT n=5, 71.43%
		AK n=4, 14.81%	PT n=1, 14.29%
			TPT n=1, 14.29%
Conduit used	GSV n=26, 76.5%		
	Prosthetic graft n=8, 23.5%		
Postop Medications			
Dual antiplatelet therapy (DAPT)	n=12, 35.3%	n=9, 33.33%	n=3, 42.86%
Single antiplatelet (SAP)	n=6, 17.6%	n=5, 18.52%	n=1, 14.29%
Aspirin/ Clopidogrel + Direct oral anticoagulant (DOAC)	n=12, 35.3%	n=10, 37.04%	n=2, 28.57%
Warfarin + SAP	n=4, 11.8%	n=3, 11.11%	n=1, 14.29%

Risk factor profile

A significant portion (52.9%, n=18) were current smokers. These were present in 70.5% (n=14) and 44.1% (n=3) of the patients of fem-pop and fem-distal bypass, respectively.

Procedural details

A predominant majority (79.4%, n=27) underwent fem-pop bypass, either above the knee (AK) or below the knee (BK), while 20.6% (n=7) had a fem-distal or pop-distal bypass. Among the patients who had distal bypasses, 71.43% (n=5) had anterior tibial artery (AT) as the target for distal anastomosis, while 28.58% (n=2) had posterior tibial artery (PT) or peronial artery (PA) as the distal target (Table 1).

Postoperative outcomes

**Figure 1:**
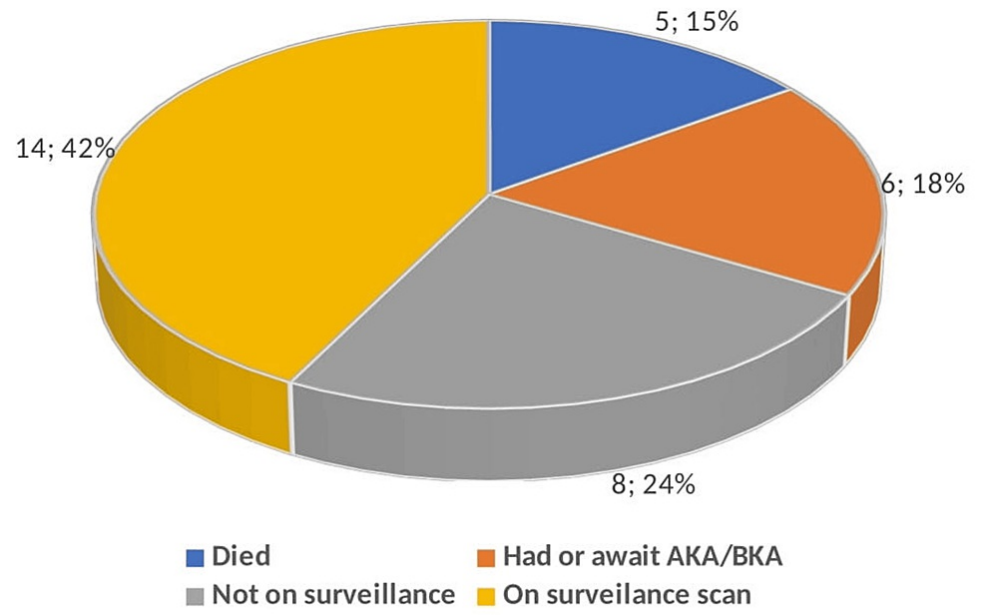
Pie chart showing the outcome of the infrainguinal bypass. AKA (Above-knee amputation), BKA (Below-knee amputation)

The study noted that 15% (n=5) of the patients passed away, and 18% (n=6) had either undergone or were awaiting major limb amputation (Figure 1).

Surveillance and graft patency

**Figure 2:**
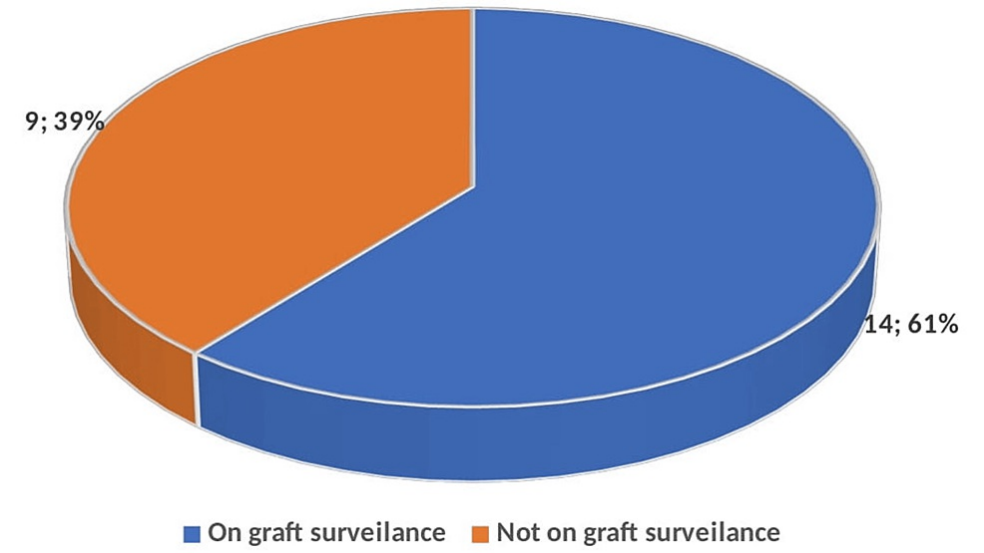
The pie chart showing the surveillance rate.

**Figure 3:**
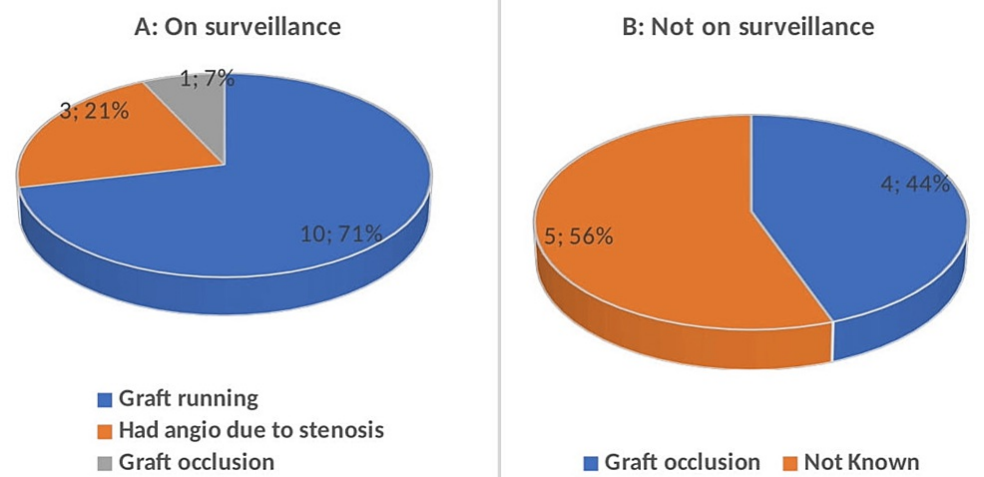
Pie charts showing graft occlusion rates in patients who were on surveillance (pie chart A) and those who were not on surveillance (pie chart B).

Sixty-one percent (n=14) of the patients were enrolled in a graft surveillance program (Figure 2). Of these, 71% (n=10) maintained functioning grafts, 21% (n=3) required angiography due to graft stenosis, and 7% (n=1) experienced graft occlusion. Forty-four percent (n=4) experienced graft occlusion, and 56% (n=5) had outcomes that remained unknown (Figure 3).

These results underscored the critical importance of postoperative surveillance and the need for stringent management of risk factors to enhance outcomes in CLTI patients undergoing infrainguinal bypass surgery. The high prevalence of risk factors such as smoking, hypertension, and diabetes also indicated the necessity for more comprehensive pre- and postoperative patient management strategies.

## Discussion

The findings of this study elucidate several key insights and implications for the management of patients with CLTI following infrainguinal bypass surgery. The high prevalence of modifiable risk factors, such as smoking, hypertension, and diabetes mellitus, in the study cohort emphasizes the need for more stringent perioperative risk factor management. This involves not only medical interventions but also patient education and lifestyle modifications. The superiority of autogenous vein grafts in terms of patency rates is reaffirmed by the study. This stresses the importance of considering autologous veins as the first choice for bypass grafts, wherever feasible.

The study demonstrates the critical role of regular postoperative surveillance, particularly using duplex scans, in the early detection and intervention of at-risk grafts. Surveillance has been shown to be instrumental in reducing the incidence of graft occlusion and improving overall graft patency. The varied outcomes and complications observed highlight the necessity for each vascular unit to develop and implement comprehensive, tailored follow-up strategies. These strategies should address the specific needs and risks of the CLTI patient population, ensuring close monitoring and prompt intervention when necessary.

The report of the Inter-Society Consensus for the Management of CLTI recommended a clinical surveillance program after infrainguinal bypass surgery consisting of interval history, vascular physical examination, and measurement of ankle-brachial index (ABI) without routine duplex ultrasound scanning (DUS) [9]. However, despite this lack of high-quality evidence, several single-centre studies have identified DUS velocity parameters that appear to be predictive of vein graft thrombosis [10]. The six-week postoperative duplex ultrasound scan is important in identifying flow abnormalities that can predict the natural history of a vein graft and the outlook for the limb in the medium term [11]. A graft surveillance program should result in a graft failure rate of less than 3% per year [12]. DUS was recommended after femoral-popliteal and femoral-tibial-pedal vein bypass grafts with surveillance intervals of approximately three, six, and 12 months and then yearly thereafter [13]. Other authors favour surveillance for at least two years [14]. Certainly, controlled randomised studies are required before any definite answer is given on the time aspect of surveillance. Some individual grafts are at greater failure risk. Mills et al. have proposed that a less intense surveillance course may be required in a low-risk group of grafts, especially for those with normal initial findings [15].

Based on the study's findings, several actionable recommendations are proposed to enhance the management and outcomes of CLTI patients' post-infrainguinal bypass. Developing and adhering to standardized protocols for infrainguinal bypass procedures, including intraoperative assessments such as on-table completion angiography, to ensure the technical success of the surgery. Implementing a mandatory surveillance program for all patients undergoing infrainguinal bypass. This should include regular duplex scanning and clinical evaluations to promptly identify and address any graft-related issues and establishing robust programs focusing on the modification of perioperative risk factors. These programs should include smoking cessation support, diabetes management, blood pressure control, and patient education on lifestyle changes. Crafting individualized care plans for each patient, taking into account their specific risk factors, comorbidities, and social circumstances. This approach will ensure a more targeted and effective management strategy. Enhancing training and education for healthcare providers in the latest techniques and guidelines for managing CLTI and postoperative care. This includes updating skills in vascular surgery, patient management, and the use of diagnostic tools such as duplex scanning and continuously collecting and analyzing data on patient outcomes post-infrainguinal bypass surgery. These data should be used to refine and improve protocols, surveillance strategies, and overall patient care.

Limitations

The limitations of this retrospective, single-centre study relate to the sparse randomized evidence. The sample size is not large. However, the basic approach of DUS surveillance of vein grafts has not changed substantially.

## Conclusions

In summary, the study underscores the need for a holistic, guideline-driven approach to the management of CLTI patients undergoing infrainguinal bypass surgery. By implementing these actions, it is anticipated that patient outcomes will improve, with reductions in graft failure rates, lower limb amputations, and enhanced overall patient quality of life.
